# Poor Prognosis in Acute Myeloid Leukemia Patients with Monosomal Karyotypes

**DOI:** 10.4274/tjh.2016.0255

**Published:** 2017-06-01

**Authors:** Junqing Xu, Baohua Huang, Xiaoqian Liu, Yuanfeng Zhang, Yinghui Liu, Liming Chen, Yanyan Luan, Nannan Li, Xiaoxia Chu

**Affiliations:** 1 Qindao University Medical College, Affiliated Yantai Yuhuangding Hospital, Department of Hematology, Yantai, China; 2 Qindao University Medical College, Affiliated Yantai Yuhuangding Hospital, Laboratory of Hematology, Yantai, China

**Keywords:** Leukemia, Monosomal karyotype, prognosis

## Abstract

**Objective:**

This study aimed to investigate the clinical characteristics and prognostic significance of monosomal karyotypes (MKs) in patients with acute myeloid leukemia (AML).

**Materials and Methods:**

We retrospectively analyzed the clinical data for 498 patients with AML, of whom 233 (46.8%) had an abnormal karyotype, including 42 with MKs (8.4%) and 70 with a complex karyotype (CK) (14.1%).

**Results:**

Patients with MKs were older (median age 62.5 vs. 52 years, p=0.003) and had lower median hemoglobin levels (62.5 vs. 77 g/L, p=0.009) and lower white blood cell counts (7.0×109/L vs. 11.7×109/L, p=0.008). Univariate analysis showed that patients with MKs or CKs had shorter overall survival than patients without these karyotypes (median survival time 7.3 vs. 26.3 months for MK, p<0.001, and 14.8 vs. 26.3 months for CK, p<0.001). In multivariable analysis for overall survival, MK and National Comprehensive Cancer Network prognostic group were the only significant factors.

**Conclusion:**

MK is an independent risk factor for poor prognosis in AML patients.

## Abstract (tr)

**Amaç:**

Bu çalışmada akut myeloid lösemi (AML) hastalarında monozomal karyotipe (MK) sahip olmanın klinik özellikleri ve prognostik önemini araştırmak hedeflenmiştir.

**Gereç ve Yöntemler:**

Retrospektif olarak 498 AML hastasının klinik verileri incelenmiştir. Bu hastalardan 233’ünde (%46,8) anormal karyotip saptanmış olup, bunların da 42’sinde (%8,4) MK ve 70’inde kompleks karyotip (KK) bulunmuştur.

**Bulgular:**

Hastalardan MK’ya sahip olanlar daha yaşlı olup (ortanca 62,5 vs. 52 yıl; p=0,003), daha düşük ortanca hemoglobin düzeyine (62,5 vs. 77 g/L; p=0,009) ve daha düşük beyaz küre sayısına (7,0×109/L vs. 11,7×109/L, p=0,008) sahipti. Ünivaryant analizde MK ya da KK’ya sahip olan hastaların diğer hastalara göre toplam sağkalımı daha düşük bulundu. (MK için ortanca sağkalım süresi 7,3 vs. 26,3 ay, p<0,001 ve CK için 14,8 vs. 26,3 ay, p<0,001). Toplam sağkalım için yapılan, multivaryant analizde ise MK ve “National Comprehesnsive Cancer Network” prognostik grubu anlamlı bulunan faktörlerdi.

**Sonuç:**

AML’li hastalarda MK kötü prognoza işaret eden bağımsız bir risk faktörüdür.

## INTRODUCTION

Acute myeloid leukemia (AML) is a malignant clonal disease originating from myeloid hematopoietic stem/progenitor cells, with high heterogeneity in terms of clinical manifestations, histopathology, cytogenetics, molecular genetics, and immunophenotypes. Accurate evaluation of the prognosis of patients with AML is important for establishing clinical therapeutic protocols. About 50%-60% of AML patients have cytogenetic abnormalities. A complex karyotype (CK) is an independent unfavorable prognostic factor in AML patients, and recent studies have demonstrated that patients with a monosomal karyotype (MK) have shorter survival than those with CKs [1,2,3]. However, MK has not been included in the traditional prognostic scoring systems. This study investigated the prognostic significance of MKs and CKs in a cohort of 498 patients with AML.

## MATERIALS AND METHODS

### Study Samples and Data Sources

The study was approved by the Ethics Committees of Yuhuangding Hospital and was conducted according to the guidelines of the Declaration of Helsinki. Due to recent wide application of cytogenetic analysis in our hospital, a total of 498 patients diagnosed with AML from July 2001 to July 2013 were included in this study. All patients were strictly reevaluated according to the 2008 World Health Organization criteria [4]. Standard daunomycin (DA) (DA at 45 mg/m^2^/day for 3 days plus cytosine arabinoside at 150 mg/m^2^/day for 7 days) or mitoxantrone (MA) (MA at 6-8 mg/m^2^/day for 3 days plus cytosine arabinoside at 150 mg/m^2^/day for 7 days) protocols were given to 452 patients as induction therapy. The clinical efficacy was evaluated according to the revised recommendations of the International Working Group for Diagnosis, Standardization of Response Criteria, Treatment Outcomes, and Reporting Standards for Therapeutic Trials in AML [5]. For patients who achieved a complete response (CR), the above two regimens were continued, or regimens based on moderate-dose cytosine arabinoside at 2 g/m^2^/day for 3 days were used for consolidation and intensification therapies. Patients without CR received MAC (mitoxantrone at 6-8 mg/m^2^/day for 3 days plus cytosine arabinoside at 150 mg/m^2^v/day for 7 days plus cyclophosphamide at 400 mg/m^2^/day on days 2 and 5) or FLAG (fludarabine at 30 mg/m^2^/day for 5 days plus cytosine arabinoside at 1-2 g/m^2^/day for 5 days plus recombinant granulocyte-colony stimulating factor at 300 µg/day for 5 days) as a second induction therapy. Forty-six (9.2%) patients were treated with palliative therapies, mainly including blood transfusion and hydroxycarbamide, to reduce white blood cells. All patients were followed up to 31 March 2014. The median follow-up time was 24 months (range: 2-145 months). The overall survival (OS) time was defined as the time from diagnosis to the last date of follow-up or the date of death.

### Cytogenetic Analysis

Cytogenetic analysis was performed at first diagnosis for all patients. Bone marrow cells were collected from all patients and cultured for 24-48 h, and then used for routine preparation of slices for banding of G fragments. Karyotypes were determined according to the International System for Human Cytogenetic Nomenclature (ISHCN 2009). One abnormal clone was defined as cells with ≥20 nuclear metakinesis phases and ≥2 cells with the same chromosome increase or structural rearrangement, or ≥3 cells with the same chromosome deletion in chromosome detection. MK was defined as ≥2 distinct autosomal monosomies or one autosomal monosomy combined with an abnormal structure in one clone [1], and CK was defined as ≥3 chromosome abnormalities in one clone [6].

### Statistical Analysis

Clinical and laboratory parameters at diagnosis or first referral were analyzed statistically using SPSS 18.0. Numerical variables were summarized as medians and ranges. Categorical variables were compared using chi-square statistics, and continuous variables were compared between different categories using Mann-Whitney U tests. Survival was measured from the date of diagnosis to the date of death or the last known follow-up and estimated using the Kaplan-Meier method. Survival data were compared using log-rank tests. Multivariate analysis was conducted using a Cox proportional hazards regression model. All p-values were two-tailed and statistical significance was set at p<0.05.

## RESULTS

### Clinical and Laboratory Characteristics of Patients with Monosomal Karyotypes

Of the 498 patients with AML, 271 (54.4%) were males and 227 (45.6%) were females, with a median age of 54 years (12-89 years). Twenty-two (4.4%) patients had a history of myelodysplastic syndrome or myeloproliferative neoplasms. All patients had analyzable chromosome karyotypes. Of the 498 AML patients, 233 (46.8%) had abnormal karyotypes, including 42 with MKs (8.4% of all patients and 18.0% of those with an abnormal karyotype) and 70 with CKs (14.1% of all patients and 30.0% of those with an abnormal karyotype). In addition, of the 42 patients with MKs, 36 (85.7%) also met the criteria for CK, while 36 of the 70 patients with CKs (51.4%) also met the criteria for MK. Compared with patients without MKs, patients with MKs were older and had lower hemoglobin levels and lower white blood cell counts. Moreover, AML patients with histories of myelodysplastic syndrome or myeloproliferative neoplasms were more likely to have MKs than de novo AML patients (p=0.038).

Of the 42 patients with MKs, 15 had one chromosome monosomy concomitant with structural abnormality and 27 (64.3%) had ≥2 chromosome monosomies. The most common monosomies were -7 (12 cases, 28.6%), -17 (9 cases, 21.4%), -20 (7 cases, 16.7%), and -5 (6 cases, 14.3%) (Table 1).

### Treatment Effect Analysis

Of the 498 AML patients, 11 died of severe pre-chemotherapeutic complications, including three patients with MKs and eight patients without MKs. Thirty-five (7.1%) patients were treated with palliative therapy. Of the 452 (90.8%) AML patients treated with induction therapy, 35 (7.7%) had MKs and 417 (92.3%) were without MKs. CR was achieved in 20% of patients with MKs compared with 61.4% of patients without MKs. Patients with MKs had significantly shorter OS than those without MKs (p<0.001). There was no difference in the outcome of patients with one monosomy combined with one structural abnormality and those with 2 monosomies (p=0.226).

### Prognostic Significance of Monosomal Karyotypes in Acute Myeloid Leukemia Patients

**Figure 1:**
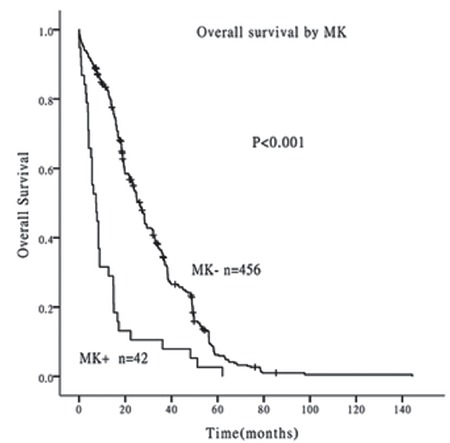
Survival curves of patients with and without monosomal karyotypes. The overall survival was poorer in patients with monosomal karyotypes than those without (median survival time 7.3 vs. 26.3 months, p<0.001). MK: Monosomal karyotype.

**Figure 2:**
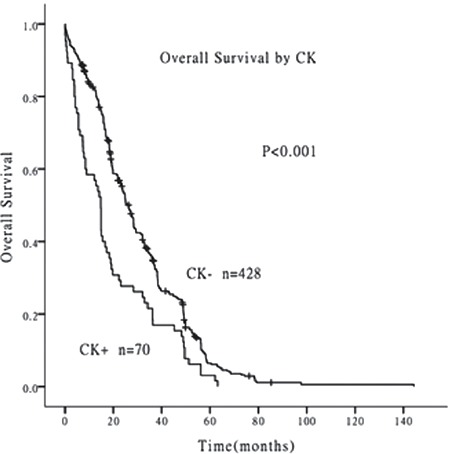
Survival curves of patients with and without complex karyotypes. The overall survival was poorer in patients with complex karyotypes than those without (median survival time 14.8 vs. 26.3 months, p<0.001). CK: Complex karyotype.

Univariate analysis indicated that OS was poorer in patients with MKs compared with those without (median survival time 7.3 vs. 26.3 months, p<0.001) (Figure 1) and in patients with CKs compared with those without (median survival time 14.8 vs. 26.3 months, p<0.001) (Figure 2). Age (p=0.017) and National Comprehensive Cancer Network (NCCN) prognostic grouping (p<0.001) were also identified as prognostic factors in AML patients. CK, MK, and NCCN prognostic group were included in a Cox regression risk model analysis, which identified MK as a prognostic factor independent of NCCN prognostic grouping in AML patients (Table 2). The results indicated that MK, rather than CK, was a risk factor for poor prognosis in AML patients.

## DISCUSSION

Breems et al. [1] previously showed that AML patients aged 15-60 years with MKs had a poorer prognosis than those with CKs, with a 4-year OS rate of only 4%. In 2010, the Southwest Oncology Group found a similar 4-year OS rate of 3% for patients with MKs among 1344 AML patients aged 16-88 years [7]. The results of the current study are consistent with those previous reports, with a median survival time and 3-year OS rate for MK+ AML patients of 7.3 months and 6.2%, respectively. The incidence of MKs in this study was 8.4%, which was consistent with the 9%-15% reported in other studies [1,2,8,9,10,11].

AML patients with MKs in this study were older and had lower white blood cell counts and hemoglobin levels than those without MKs, consistent with the results of Kayser et al. [2] and in line with the age-related increase in cytogenetic abnormalities. However, the mechanisms responsible for MKs are still unclear, though they may be associated with deletions or mutations in potential multiple drug resistance genes and *TP53* [12,13,14]. MK + AMLs were significantly associated with *TP53* alterations, which appear to be one molecular basis for this purely descriptive cytogenetic subset [13].

Patients with MKs have a poor response to routine chemotherapy, a higher recurrence rate, and shorter long-term survival. However, some studies found that high-dose cytosine arabinoside-based chemotherapeutic regimens could improve long-term survival in MK + AML patients to some extent [15,16]. Fang et al. [17] also suggested that allogeneic hematopoietic stem cell transplantation could increase the 4-year disease-free survival rate of MK + AML patients to 25%. Although this was still lower than the 56% reported for MK - AML patients, it was obviously higher than the 3-9% for patients receiving chemotherapy alone. Subsequent studies also confirmed that transplantation could improve long-term survival in MK + AML patients, but it was associated with disadvantages such as a high recurrence rate and short median time to recurrence [2,18,19].

## CONCLUSION

In conclusion, this study confirmed that MK was a poor prognostic factor in patients with AML independent of age, CK, and NCCN prognostic grouping. When combined with cytogenetics and NCCN prognostic grouping, MK status could further improve the prognostic classification accuracy in patients with AML.

## Figures and Tables

**Table 1:**
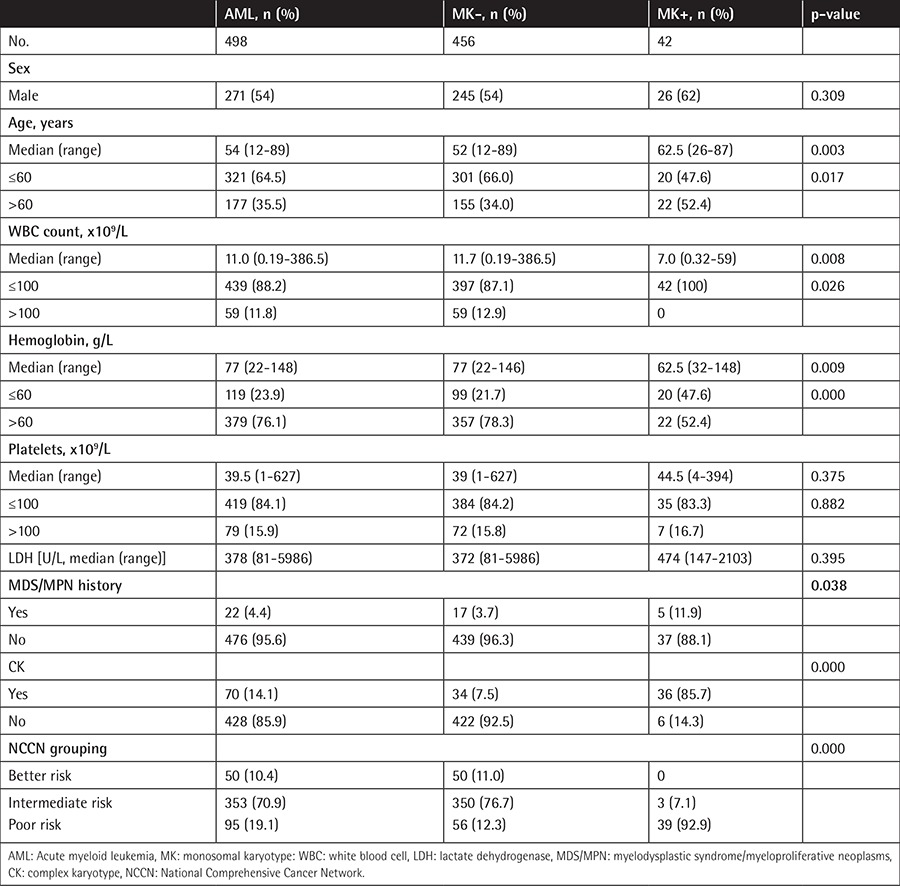
The clinical features of patients with acute myeloid leukemia

**Table 2:**
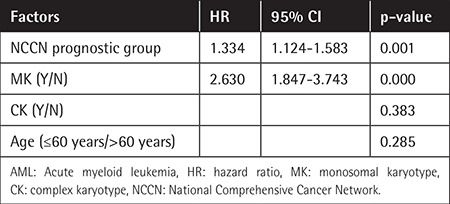
Analysis results using a Cox regression risk model in patients with acute myeloid leukemia.
